# DNA unwinding mechanism of a eukaryotic replicative CMG helicase

**DOI:** 10.1038/s41467-020-14577-6

**Published:** 2020-02-04

**Authors:** Zuanning Yuan, Roxana Georgescu, Lin Bai, Dan Zhang, Huilin Li, Michael E. O’Donnell

**Affiliations:** 1 Structural Biology Program, Van Andel Institute, Grand Rapids, MI USA; 2 Howard Hughes Medical Institute, Chevy Chase, MD USA; 3 DNA Replication Laboratory, The Rockefeller University, New York, NY USA

**Keywords:** DNA, Enzyme mechanisms, Cryoelectron microscopy

## Abstract

High-resolution structures have not been reported for replicative helicases at a replication fork at atomic resolution, a prerequisite to understanding the unwinding mechanism. The eukaryotic replicative CMG (Cdc45, Mcm2-7, GINS) helicase contains a Mcm2-7 motor ring, with the N-tier ring in front and the C-tier motor ring behind. The N-tier ring is structurally divided into a zinc finger (ZF) sub-ring followed by the oligosaccharide/oligonucleotide-binding (OB) fold ring. Here we report the cryo-EM structure of CMG on forked DNA at 3.9 Å, revealing that parental DNA enters the ZF sub-ring and strand separation occurs at the bottom of the ZF sub-ring, where the lagging strand is blocked and diverted sideways by OB hairpin-loops of Mcm3, Mcm4, Mcm6, and Mcm7. Thus, instead of employing a specific steric exclusion process, or even a separation pin, unwinding is achieved via a “dam-and-diversion tunnel” mechanism that does not require specific protein-DNA interaction. The C-tier motor ring contains spirally configured PS1 and H2I loops of Mcms 2, 3, 5, 6 that translocate on the spirally-configured leading strand, and thereby pull the preceding DNA segment through the diversion tunnel for strand separation.

## Abstract (web summary)

The DNA duplex is known to be split apart in a steric exclusion manner during replication, but the specific mechanism has remained unclear. Here the authors present a cryo-EM structure of a eukaryotic replicative CMG helicase on forked DNA, revealing the mechanism of DNA unwinding.

## Introduction

**Fig. 1:**
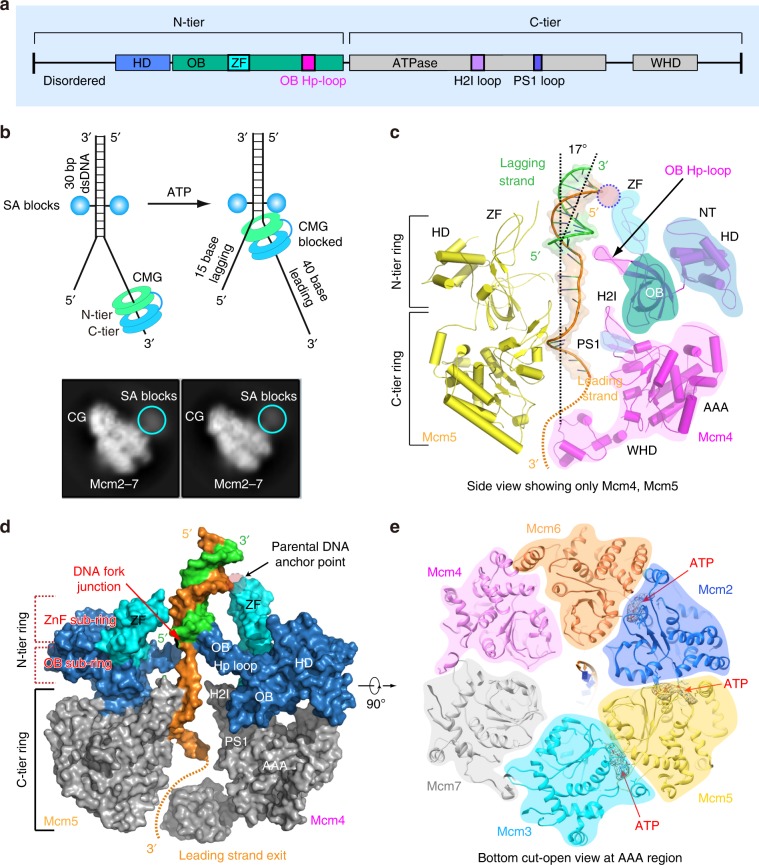
The parental dsDNA enters the zing finger region at the N-tier ring of CMG and is in-line with the unwound leading ssDNA. **a** A sketch of the general domain architecture of a typical Mcm protein, highlighting the hairpin loop in the OB domain (OB Hp-loop), and the H2I and PS1 loops in the AAA+ domain. HD: N-terminal helical domain, ZF: Zinc finger domain that is embedded in the OB domain, WHD: C-terminal winged helix domain. **b** Top panel: a sketch for the double streptavidin labeled forked DNA used to stall CMG in the presence of ATP. Bottom panel: two selected 2D class averages of the CMG stalled by the double streptavidin whose densities are visible. These averages are also shown in Fig. S1b. **c**, **d** Two side views of CMG–forked DNA complex in cartoon **c** and surface **d** views. Only Mcm4 and Mcm5 are shown to provide an unobstructed view of the forked DNA and the major structural features of a typical Mcm protein, as labeled in Mcm4. **e** A bottom view of Mcm2-7 that is cut open at the AAA+ motor ring. Cryo-EM densities for the three tentatively assigned ATP molecules are shown as gray mesh, superimposed with the atomic model of ATP in sticks.

Replicative helicases of all cell types are hexameric rings composed of a N-tier ring of the NTD domains and a C-tier ring^1^. The 11-subunit eukaryotic helicase CMG (Cdc45, Mcm2-7, GINS) contains a hexameric Mcm2-7 motor ring^1–4^. The NTD ring of the Mcm2-7 motor is further divided into an upper sub-ring of six zinc finger (ZF) domains and a lower sub-ring composed of six oligosaccharide/oligonucleotide-binding (OB) domains that bind single-stranded DNA (ssDNA)^5^. The C-tier contains the motors with ATP sites located at subunit interfaces^6,7^. The ATP-binding site of bacterial helicases is based on the RecA fold, while eukaryotic helicase ATP sites are based on the AAA+ fold^1,5,7,8^. While bacterial replicative helicases travel 5′–3′ with the C-tier motors leading the way, eukaryotic helicases track 3′–5′ on DNA with the N-face in front, pushed by motors in the C-tier^1,2,5^. The way in which helicases engage ssDNA in the motor domains has been documented for several different hexameric replicative helicases and involve binding to loops in the ATPase domains^1,5,9–16^. In eukaryotic viruses the main loop is referred to as PS1 (e.g. BPV viral E1 and SV40 T-antigen of superfamily 4 helicases); while archaeal MCMs and eukaryotic Mcm2-7 of CMG (superfamily 6 helicases) contain the pre-sensor 1 (PS1) loop and also a helix 2 insertion (H2I) loop of unknown function (Fig. 1a). The PS1 loops are proposed to pull the ssDNA such that the parental duplex splits at the top of the helicase, a process known as the steric exclusion mechanism of helicase action, because one strand is excluded from the central channel while the other strand is pulled through the central channel^17^. In simple terms, the helicases act as a moving wedge to separate the strands of dsDNA. However, *Saccharomyces cerevisiae* CMG has a wide central channel and easily accepts dsDNA^18^. Hence details of how steric exclusion actually splits the strands rather than bringing them both into the central channel of the helicase are unknown, as high-resolution structures of helicases with bound forked DNA are lacking.

Our earlier study of the budding yeast *S. cerevisiae* CMG bound to a forked DNA demonstrated that the parental duplex enters the N-tier of CMG a short distance^19^, which was different from the classic steric exclusion models^2,7^. We previously used a dual streptavidin block on DNA to stall the CMG unwinding at a fork in the presence of ATP and determined a medium-resolution cryo-EM structure of CMG on forked DNA^19^. While this study revealed the N-first orientation of CMG during DNA tracking, the 6.1-Å resolution was too low to resolve the key structural features critical for DNA unwinding^19^. In the cryo-EM study of the DNA fork-containing CMG in the presence of ATPγS and Pol ε, the average resolution reached about 5 Å^12^. However, there was a long density gap between the parental dsDNA region in the N-tier and the leading strand DNA in the C-tier motor ring; in other words, the DNA fork junction region was also not visualized in that study. Therefore, the primary motivation of this manuscript is to visualize the forked DNA junction structure at atomic or near atomic resolution—high enough resolution to define a precise mechanism of dsDNA unwinding by a eukaryotic helicase performing steric exclusion, that could otherwise draw both DNA strands through the central channel. We analyzed a CMG–Mcm10 complex because Mcm10 binds CMG tightly and can be isolated as a CMG–Mcm10 complex^20^, and because Mcm10 contains a DNA-binding module that likely underies its observed ability to facilitate DNA binding of CMG-Mcm10^21–25^.

## Results

### The structure of CMG−forked DNA in the presence of Mcm10

Mcm10 binds tightly to CMG^20,26^, enhances CMG binding to forked DNA^18^, and stimulates the rate and processivity of CMG^20^. Therefore, in the current work, we assembled reconstituted purified CMG–Mcm10 on the double-streptavidin stalled forked DNA and carried out cryo-EM analysis of the assembled product (Fig. 1b). We found that Mcm10 increased the percentage of CMG–forked DNA particles by a factor of 2 compared to that observed in our earlier study of CMG–forked DNA in the absence of Mcm10^19^. We went on to determine a 3.9-Å cryo-EM structure (Fig. 1c–e, Supplementary Figs. 1–5, Table 1, Supplementary Movie 1). To our surprise, no Mcm10 density was observed in the 2D class averages or 3D map, suggesting that Mcm10 has multiple conformations or binds at multiple sites and becomes averaged out in the 3D reconstruction process. It is also possible that Mcm10 dissociates from CMG upon freezing the sample on the cryo-EM grid. Because Mcm10 density is not observed, we will therefore refer to our 3D reconstructed structure as CMG–forked DNA in this manuscript, although Mcm10 may be present. In the new structure, the parental dsDNA enters CMG with an angle essentially the same as in the previous lower resolution structure in the absence of Mcm10 (Fig. 1c, d). We suggest that Mcm10 binding does not alter the in-line configuration of the parental dsDNA and the unwound leading ssDNA. Therefore, the DNA configuration in the eukaryotic replisome is likely different from the T7 replisome. This different orientation, in-line vs. perpendicular, appears important to the ease of unwinding^27^ and may contribute to the 10–100 fold different velocities of unwinding between eukaryotic and bacterial replicative helicases.

**Table 1:** Cryo-EM 3D reconstruction and refinement of the CMG–forked DNA complex.

	CMG–forkDNA
*Data collection and processing*	
Magnification	130,000
Voltage (kV)	300
Electron dose (e^−^/Å^2^)	80
Under-focus range (μm)	1.5–2.5
Pixel size (Å)	1.029
Symmetry imposed	C1
Initial particle images (no.)	718,903
Final particle images (no.)	162,550
Map resolution (Å)	3.9
FSC threshold	0.143
Map resolution range (Å)	3.5–5.0
*Refinement*	
Initial model used (PDB code)	3jc5
Map sharpening *B* factor (Å^2^)	−202
Model composition	
Non-hydrogen atoms	41,394
Protein and DNA residues	5016
Ligands	3
*R.m.s. deviations*	
Bond lengths (Å)	0.018
Bond angels (°)	1.43
*Validation*	
MolProbity score	2.18
Clashscore	9.94
Poor rotamers (%)	0.38
*Ramachandran plot*	
Favored (%)	86.71
Allowed (%)	13.19
Disallowed (%)	0.1

The structure contained densities for three nucleotides in the binding pockets of Mcm2, Mcm3, and Mcm5, the right half of the Mcm ring embraced by Cdc45 and the GINS tetramer (Fig. 1e, Supplementary Fig. 6). At the current resolution, the identities of these nucleotides are not absolutely certain, but the features are most consistent with ATP. The nucleotide-binding pocket of Mcm7 had a weak density, suggesting a partial occupancy by an ATP or ADP (Supplementary Fig. 6). This is consistent with the three-nucleotide binding in the earlier study, although the identities of the nucleotides were undetermined in the previous low-resolution work^19^. The current structure clearly shows that only four subunits interact with the leading strand ssDNA in the C-tier ATPase motor ring: Mcm3, Mcm5, Mcm2, and Mcm6, while the Mcm4 and Mcm7 subunits do not bind DNA in the ATPase region (to be described below). Therefore, the overall structure underscores the asymmetry in the translocating motor ring in terms of ATP binding as well as in DNA binding. This property of the eukaryotic helicase seems to be different from the archaeal MCM hexamer as observed in the recent crystal structure of a mutant SsoMCM in which the linker between the N-tier and C-tier rings is shortened but the helicase is nevertheless active^14^. In such modified archaeal MCM hexamer structure, three Mcm proteins bound to ADP and three remaining subunits bound to ADP-BeF_3_, but all six proteins made contact with the 12-base ssDNA in the central channel.

### dsDNA enters CMG to the boundary of the ZF and OB sub-rings

CMG, in the context of a full replisome in *Xenopus* extracts, is demonstrated to act by a steric exclusion process, in which the DNA is split before entering the helicase^17^. Thus, it was somewhat unexpected that dsDNA enters CMG a short distance at the ZF region, as observed in the CMG–forked DNA structure^19^ as well as in a subsequent structure of CMG−Pol ε−ATPγS on the forked DNA^12^. The ZF domains of CMG project from the extreme N-face of CMG, even though the sequence of a helical domain (HD) or A-domain in the NTD of MCM subunits comes earlier in the primary sequence (Fig. 1a), the ZF and an OB fold are intertwined in the primary sequence and come directly after the A-region (Fig. 1c, d)^28^. In the current 3.9-Å structure, the ZFs project from the N-tier of Mcm2-7 and encircle and contact the dsDNA (Fig. 1c, d). Therefore, both the future leading and lagging strand DNA, while still paired in the parental duplex, are in fact contacted by CMG before their separation into single strands. However, the binding is not extensive, and only proceeds to the floor of the ZFs, after which the strands are separated. This separation point will be examined further in the next section. Physical interaction of CMG in the dsDNA region may explain why bulky groups that are held close, within 6–10 Å, to the duplex inhibited helicase activity^20^.

### The OB loops form a barrier that blocks the lagging strand

**Fig. 2:**
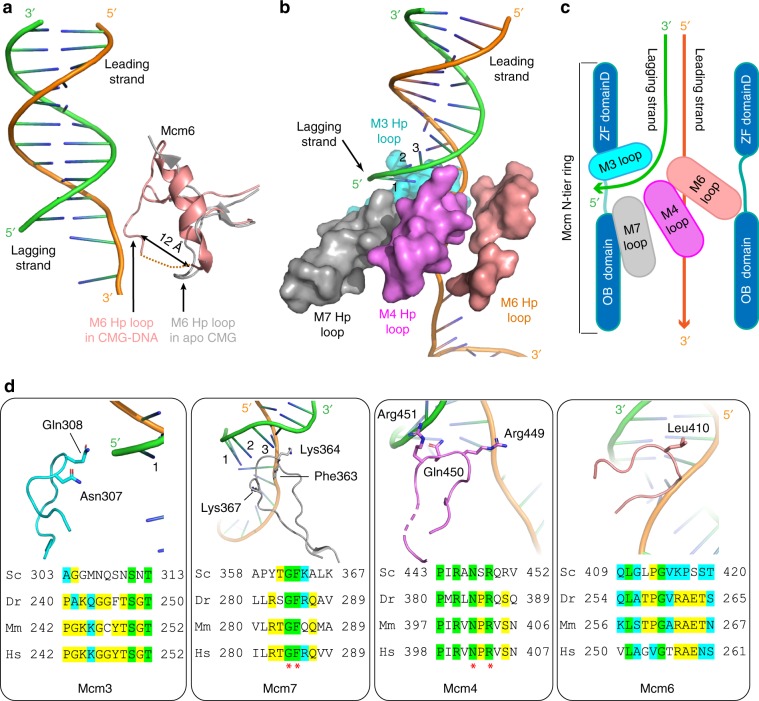
Interactions between four OB Hp-loops and DNA at the fork junction. **a** The Mcm6 OB hairpin loop undergoes a major conformation change upon binding to DNA. In the absence of DNA (gray) this region is largely disordered. In the presence of DNA (wheat), this region forms an α-helix that projects the OB Hp loop ~12 Å towards the center to interact with the DNA fork. **b** The four OB Hp loops surrounding the forked DNA. The OB folds of Mcm3,7 direct the lagging flap such that it proceeds through the zinc fingers of Mcm3,5. **c** A sketch showing that the three OB Hp loops (of Mcm4, Mcm6, and Mcm7) spiral below thus blocking the lagging strand, while one OB Hp loop (of Mcm3) is above the lagging strand and may prevent the lagging strand from backing out from the top entry of the Mcm2-7 channel. As in panel **b**, the OB loops direct the lagging strand throught the zinc finger domains between Mcm3,5 (not shown here). **d** Close-up views of the four fork-interacting OB Hp loops with key residues shown in sticks. Below each panel is shown a sequence alignment of the OB Hp loop from Sc—*Saccharomyces cerevisiae*, Dr—*Danio rerio*, Mm—*Mus musculus*, and Hs—*Homo sapiens*. Invariant residues are highlighted in green, identical residues in yellow, and similar residues in cyan. Number shown next to the lagging ssDNA is counted from the last visible 5’ end of the lagging strand.

At the improved resolution of 3.9 Å, new DNA contacts in the N-tier ring reveal how the dsDNA is split apart during the ATP-driven translocation by the C-tier motors. The region in the Mcm6 OB domain from residue 403 to 453 undergoes a dramatic conformational change as compared to the apo CMG (Fig. 2a). In the absence of DNA these Mcm6 OB loop residues adopt a structure with two β-strands that is a substantial distance from the central channel of CMG. Upon helicase activity on forked DNA with ATP in the current structure, this Mcm6 OB loop region changes to an α-helix that projects the hairpin (Hp) loop 12 Å towards the central channel to interact with the DNA forked junction region. This Mcm6 OB Hp loop, together with OB Hp loops of Mcm4 and Mcm7, form a continuous and slanted barrier below the lagging strand (Fig. 2b, Supplementary Fig. 7). Thus, as the leading strand is pulled from below in the C-tier motor, the 5′ lagging strand flap at the fork is blocked by this barrier and can no longer move downwards along with the leading strand. Instead, the lagging strand flap is diverted sideways, and it is in this manner that the 5′ flapped duplex DNA is unwound and separated to the outside of CMG for steric exclusion unwinding (Fig. 2c).

Interestingly, unlike the OB Hp loops of Mcm6, Mcm4, Mcm7 that are below the lagging strand, the OB Hp loop of Mcm3 contacts the unwound lagging strand from above (Fig. 2b–d). This strategic position prevents the lagging strand from making a U-turn to back out of the central chamber from the top entry. Therefore, the four OB Hp loops together appear to form a diversion tunnel, with three OB Hp loops of Mcm7, 4, and 6 forming the lower wall, and the single OB Hp loop of Mcm3 forming the upper wall, thereby guiding the lagging strand towards the side to exit the helicase.

Comparison of sequences of the four OB Hp loops of Mcm3, Mcm4, Mcm6, and Mcm7 indicate some degree of conservation among eukaryotes (Fig. 2d). The Mcm7 OB Hp loop contains two lysine residues (Lys-364 and Lys-367) and one conserved and bulky phenylalanine (Phe-363) near the fork junction. The Mcm4 OB Hp loop contains two conserved arginine residues (Arg-449 and Arg-451) and one conserved polar residue (Gln-450) proximal to the junction. However, residues of the OB Hp loops of Mcm3 and Mcm6 that likely interact with the forked junction are less conserved, involving polar and small hydrophobic residues. The density and models for all the DNA interacting loops of CMG are shown in Supplementary Fig. 7.

### A possible exit port for the lagging strand DNA

**Fig. 3:**
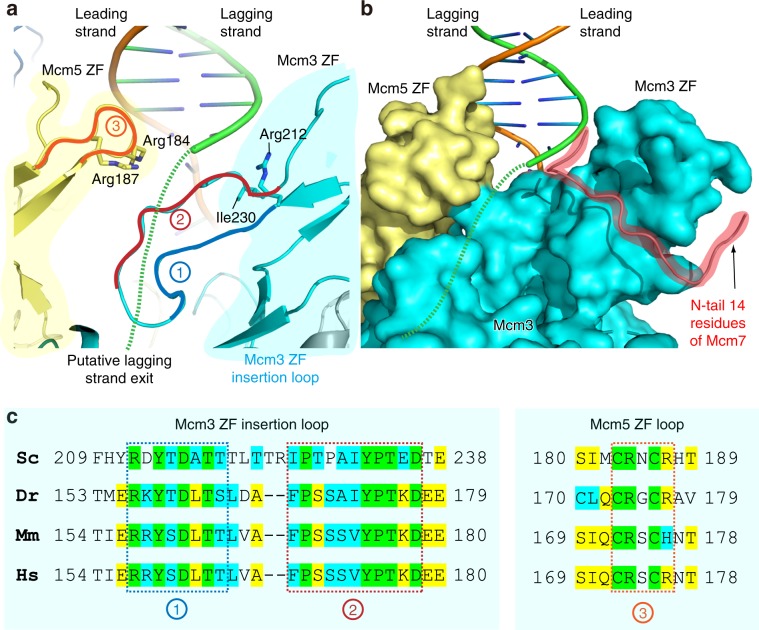
A putative exit groove of lagging strand DNA between ZF domains of Mcm3 and 5. **a** Close up of the DNA fork within the ZF regions, with the position of cryo-EM density loss of the lagging strand (dashed green curves) positioned toward the viewer. This density loss occurs at a groove between ZF domains of Mcm3 and 5. The floor of the groove is lined by a long hairpin loop (labeled by 1 and 2 inside red circles) of Mcm3 ZF domain, which has the unique and conserved feature among eukaryotic CMGs of lacking a Zn atom. A loop in the Mcm5 ZF domain (labeled by “3” in a red circle) forms the left wall of the groove and projects two Arg residues towards the putative lagging strand groove. **b** The gap between ZF domains of Mcm3 and 5 is sustained by the ordered N-terminal 14-residue peptide of the neighboring Mcm7, shown in cartoon and highlighted in red shade. This peptide forms the right wall of the groove. **c** Sequence alignment of the groove-forming loops of the ZF domains of Mcm3 and Mcm5. Sc: *Saccharomyces cerevisiae*, Dr: *Danio rerio*, Mm: *Mus musculus*, and Hs: *Homo sapiens*. Invariant residues are highlighted in green, identical residues in yellow, and similar residues in cyan.

In the current structure, the density of the lagging strand past the fork junction is lost at the base between the ZF domains of Mcm3 and Mcm5. Several structural features suggest that this region forms a possible exit path for the unwound lagging strand. Firstly, the Mcm3 subunit lacks a Zn atom, a conserved modification of Mcm subunits in eukaryotes^1,5^, and likely explains why the ZF domain of Mcm3 appears collapsed in the structure compared to those of other Mcm subunits that contain a Zn atom (Fig. 3a, b). Secondly, a sizable gap between ZF domains of Mcm3 and Mcm5—required for the potential passage of the lagging strand—is maintained by the N-terminal loop (aa 1–14) of the neighboring Mcm7, which is well ordered and reaches over to interact with the Mcm3 ZF domain. The N-terminal 100–200 residues of an Mcm protein is typically disordered (Fig. 1a); thus it is unique and unexpected that the extreme N-terminus of Mcm7 is well ordered and reaches over to interact with, and stabilize the Mcm3 ZF domain (Fig. 3a, b). Thirdly, the floor of the gap between the ZF domains of Mcm3 and Mcm5, formed by an extended insertion hairpin loop of the Mcm3 ZF domain is well conserved, as highlighted by blue and red boxes in Fig. 3c. Furthermore, on the left side of the gap, the Mcm5 ZF domain appears to project two conserved arginine residues (Arg-184 and Arg-187) towards the gap that likely interacts with the lagging strand (Fig. 3a, c). Taken together, the lagging strand is likely shunted out of CMG after entering the ZF “tower” over the collapsed floor between the ZF domains of Mcm3 and Mcm5, preventing entry into the bona fide central channel of CMG.

### The leading ssDNA appears stretched at the fork junction

**Fig. 4:**
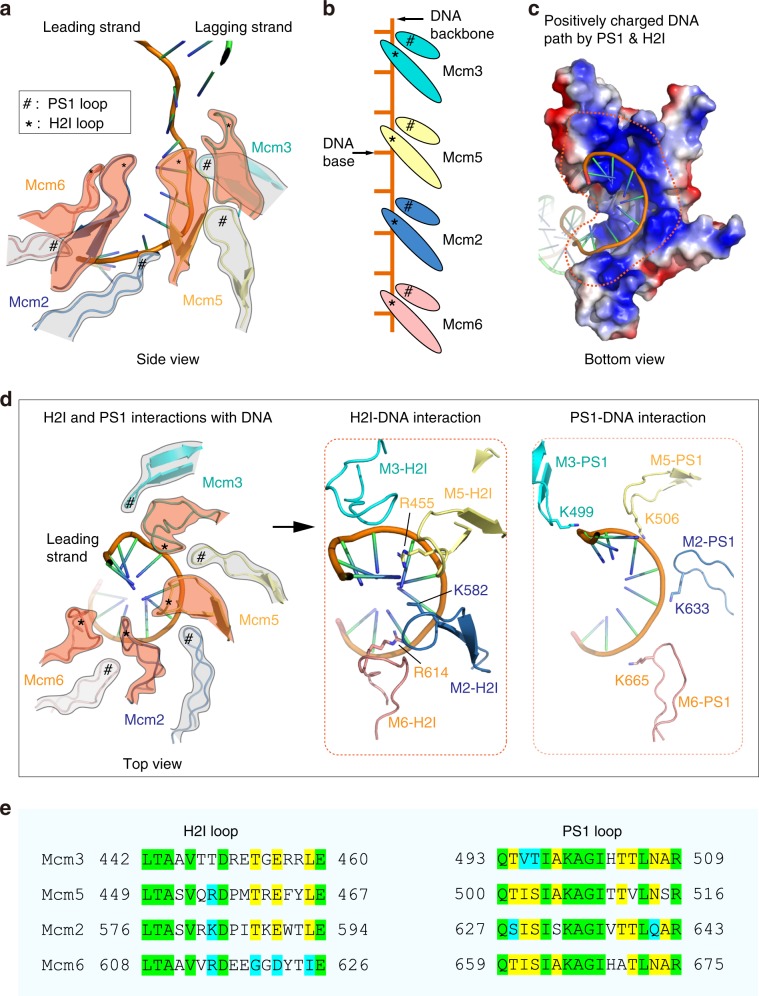
The leading ssDNA inside the C-tier motor ring of CMG helicase. **a** A side view of the DNA in the CMG chamber. The DNA-translocating PS1 and H2I loops of only four Mcm subunits (Mcm6, Mcm2, Mcm5, and Mcm3) spiral around and make contact with the leading ssDNA. **b** An illustration showing that among the four Mcm subunits engaging the DNA, each PS1 loop binds two phosphates along with an H2I loop binding over a base. The DNA-translocating loops rise two bases per Mcm subunit. **c** A bottom view showing the positively charged spiral path of the leading ssDNA formed by the PS1 and H2I loops of Mcm2, Mcm3, Mcm5, and Mcm6. **d** A top view of the PS1 and H2I loops in Mcm 3, 5, 2 and 6 engaging the spiral leading ssDNA. The middle and right panels show the DNA-interacting positively charged residues of H2I (middle) and PS1 (right). **e** Sequence alignment of the H2I and PS1 loops among the four DNA-interacting Mcm subunits. Invariant residues are highlighted in green, identical residues in yellow, and similar residues in cyan.

Under relaxed and normal conditions, the conformation of ssDNA is more condensed than dsDNA because it has many more degrees of freedom and can take on various structures that dsDNA does not have^29^. This is clearly not observed for the leading ssDNA within the N-region of CMG. The unwound leading strand has relatively weak density in this region but is nearly linear (Figs. 1c, d and 4a). The motors of CMG are in the C-tier, below the N-tier during duplex DNA unwinding. Thus, one may deduce that that the CMG motors in the C-tier pull the leading strand DNA such that the parental lagging strand is forced against the slanted OB loop barrier leading to the diversion of the lagging strand towards the side port as discussed above. Importantly, there is little interaction between the N-tier linear segment of the leading strand and the helicase. Thus, the nearly linear conformation of the leading ssDNA in the N-tier channel may be explained by the pulling force of the motors in the C-tier below. This structural feature is consistent with the steric exclusion model, in which DNA is separated at the top N-tier of the helicase—between the ZF ring and OB ring—and not in the middle between the N-tier and C-tier.

### Binding of the leading ssDNA to the C-tier motor domains

The AAA+ motif of the CMG ATP site (SF6 helicase) contains loops that bind to DNA, referred to as PS1 and H2I^8^. The PS1 loops are thought to be the main loops that act in the ATP-driven translocation complex, because the related eukaryotic viral BPV E1 and SV40 T-antigen helicases, in the SF4 class, are AAA+ helicases that contain PS1 loops but lack H2I loops^8^. Although each of the six MCM proteins has a PS1, only four PS1 loops interact with the leading ssDNA in our structure (i.e. the PS1 loops of Mcm3, Mcm5, Mcm2, and Mcm6 (Fig. 4a–e)). Each of the four PS1 loops binds two phosphodiester backbone links (Fig. 4b). This two-phosphodiester-per-PS1-binding mode also applies to the bacterial DnaB and phage T7 helicase, as revealed by recent high-resolution structures^8,11,30^. This has important implications in generalizing helicase activity from bacteria to eukaryotes as explored further in the “Discussion” section. In contrast, the H2I element, unique to the SF6 class to which CMG belongs, is observed in the current structure to interact with a nucleotide base located between the two phosphodiester bonds bound by the PS1 loop, and not the phosphate backbone (Fig. 4d). The H2I loop of Mcm5 is largely disordered in apo CMG in the absence of ATP and DNA, but this loop becomes much better ordered and undergoes a conformation change in the presence of the forked DNA, in which a peptide segement preceding the H2I loop folds into a short helix (Fig. 2a, Supplementary Fig. 7). The four H2I loops join the four PS1 loops to form a highly positively charged and well-conserved spiral path for the DNA spiral in the motor region (Fig. 4c, e). In our CMG–forked DNA structure, the manner in which the four H2I loops interact with nucleotide bases is essentially the same, and implies that the H2I interaction to DNA is an important element of DNA translocation.

## Discussion

The improved resolution of 3.9 Å has provided an unprecedented visualization of a fork junction in a replicative DNA helicase. The structure shows that the parental duplex enters the ZF sub-ring, and the strand separation occurs at the bottom of the ZF sub-ring and the beginning of the OB sub-ring. The unwound leading ssDNA traverses the OB sub-ring almost linearly in the N-tier but spirals through the C-tier motor ring before emerging from the C-face of the CMG helicase. Our earlier studies revealed that bulky blocks on the lagging strand inhibited the CMG helicase, consistent with structural studies showing that dsDNA enters the helicase^20^. However, use of >40-Å multi-PEG spacer linkers to tether bulky groups to the lagging strand did not inhibit CMG^30^. We presume that these long linkers enable the lagging strand base to reach the fork junction inside CMG and can be unwound while keeping the steric block outside of the surface of the CMG helicase. The height of the ZF collar is about 30 Å, making such scenario possible.

The dsDNA entering CMG in the presence of ATP is similar to the recent CMG–Pol ε–forked DNA structure using ATPγS^12^. Hence, the evidence would suggest that DNA enters CMG in-line, with the parental duplex being held by the ZF collar, unobstructed until reaching to the OB sub-ring. The ZF collar in the eukaryotic CMG apparently sets the approaching angle of the parent DNA and may further protect the fork from other helicases like Pif1 or Rrm3 during normal replication.

**Fig. 5:**
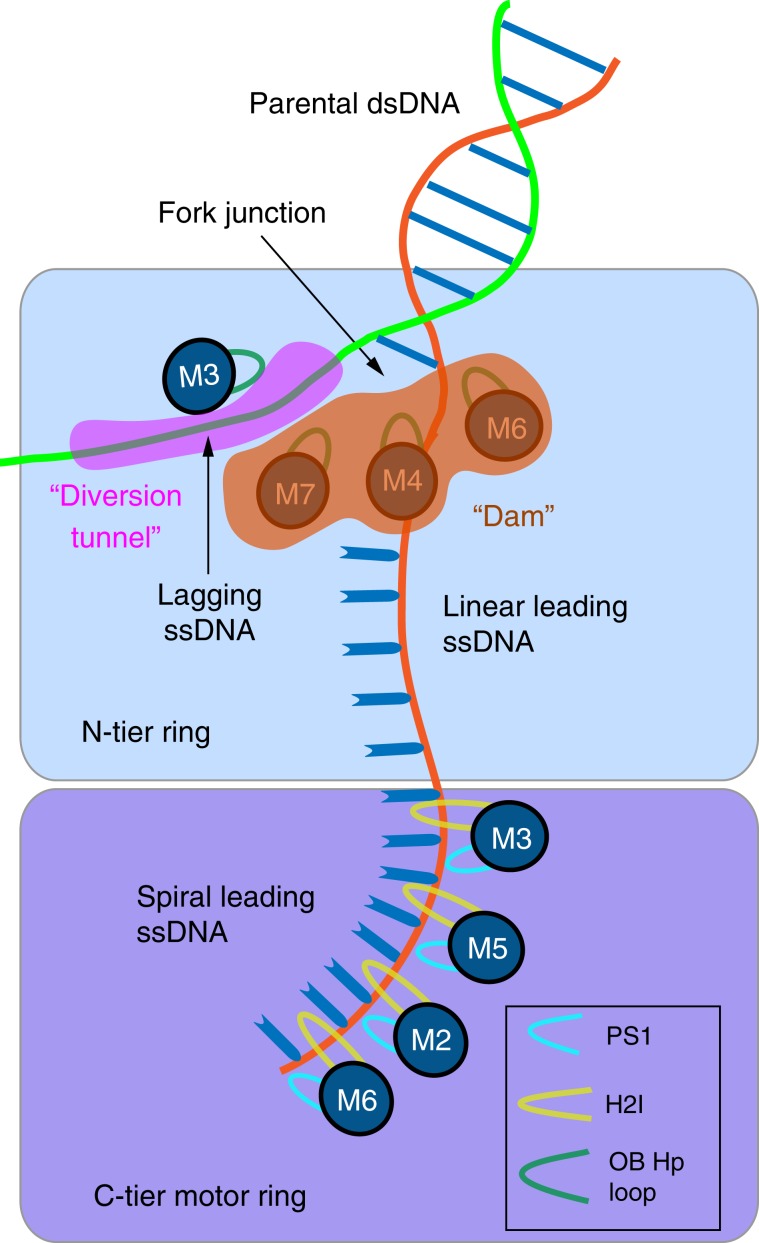
Model for DNA unwinding in a steric exclusion CMG helicase derived from this study. OB Hp loops in the NTDs of Mcms7, 4 and 6 form a block, or dam for incoming 5’ tailed DNA, and the OB Hp loop in the NTD of Mcm3 forms the top of a diversion tunnel, or channel for the displaced lagging 5’ strand that travels between the zinc fingers of Mcm3 and 5 at the N-tier surface of CMG. The PS1 and H2I loops in the C-tier motor domain pull the DNA through the diversion tunnel in the N-tier for the steric exclusion process of DNA unwinding. See text for details.

The term “steric exclusion” implies there is no specific unwinding element, other than the fact that one strand of the duplex cannot fit in (i.e. is sterically excluded from) the central channel of the helicase. However, we have shown previously that *S. cerevisiae* CMG can traverse dsDNA^18^. The current study clarifies the detailed interactions between the DNA fork junction and CMG, leading us to propose the following “diversion-tunnel” model for steric exclusion DNA unwinding by CMG (Fig. 5). In this model, the three OB loops of Mcm7, 4, and 6 form a dam just below the lagging strand at the fork nexus, and the OB loop of Mcm3 located above the lagging strand, together with those of Mcm4, 7, and 6, form the diversion tunnel via which the lagging strand is guided sideways, and may exit the CMG chamber via a gap between the ZF domains of Mcm3 and Mcm5. The driving force for the duplex unwinding is then the pulling force of the C-tier motors on the leading strand DNA from below.

Our model is different from the widely anticipated “separation pin” model of steric exclusion, in which a specific structural element is responsible for DNA unwinding, which would in turn predict a highly conserved structural element or amino acid sequence among all eukaryotic helicases. A highly conserved sequence such as this has not been identified and may not exist. In our “diversion-tunnel” model, the structural elements—the OB loops that actually form the dam and the tunnels—are largely structural and require neither specific amino acid sequences nor specific interactions between the forked DNA and the unwinding apparatus.

We note that only four of the six subunits of CMG bind ssDNA in the six AAA+ modules, similar to our lower resolution study^19^ and to the CMG-Pol ε-fork-ATPγS study^12^. In contrast, the homo-hexameric replicative helicases appear to bind ssDNA with all six of the AAA+ domains^5,14^. Why only four, but not all six, PS1 loops in CMG simultaneously pull on the leading ssDNA is currently not understood. It is possible that we have captured just a single pose and the two unbound PS1 loops will engage the leading ssDNA during a full translocation cycle. This possibility is supported by the fact that the DNA-interacting PS1 loops are located on Mcm2, Mcm3, Mcm5, and Mcm6 in the ATP-bound structure, but are located on Mcm4, Mcm6, and Mcm7 in the ATPγS-bound structure of Drosophila CMG^31^. It is also possible that just 4 subunits form a “rotational” staircase model, in which the four subunits alternate in succession, rather than 6 subunits that form a complete ring.

The two-phosphates-per-PS1 DNA-binding mode observed in CMG is shared with the *E. coli* DnaB helicase and the phage T7 helicase^8,11^, and this DNA binding mode is further reported in the *E. coli* clamp loader AAA+ ring ATPases^32^. Thus, the binding of two phosphodiester bonds per subunit in a AAA+ motor may often be used by replicative helicases and possibly other DNA helicases and translocases. However, helicases that bind only one phosphodiester link per translocation loop have also been described, such as the *E. coli* Rho factor and Bovine Papilloma Virus E1 helicase^10,15^. It is currently unclear why some helicases translocate on DNA one base at a time, while others like CMG translocate two bases per step.

The function of H2I is less well understood. Although the H2I motif is not present in SF4 AAA+ helicases, this motif is required for activity in the archaeal MCM helicase^33^. The H2I loops clamp down on the leading strand in the crystal structure of archaeal MCM^34^. In our CMG structure, each of the four H2I loops of Mcm3, Mcm5, Mcm2, and Mcm6 contacts a nucleotide base, in sync with the four PS1 loops of the same Mcm subunits contacting the phosphodiester backbone, as if the H2I loops play a secondary yet essential role in support of DNA translocation by the PS1 loops.

It is generally accepted that the C-tier motors move like a staircase in homo-hexameric helicases^2,7,10,16^. How C-tier motors translocate DNA in the eukaryotic CMG has been largely unknown because each Mcm subunit is a distinct protein and in vitro mutational studies suggest that only two sites are essential for helicase activity^3^. However, all the Mcm subunit ATP Walker A sites are required for robust cell viability^35,36^. As mentioned above, staircasing would not necessarily require all six subunits, and that a consecutive order of firing in adjacent subunits would amount to a staircase process.

We and others previously suggested at least two non-staircasing motions that could move DNA for CMG translocation: either the C-tier and N-tier move relative to one another^5,31,37^, or two C-tier motor subunits, Mcm2 and 5, move relative to one another in an inch-worming mechanism^5,37^. However, the C-tier/N-tier movement is thus far only observed in the apo CMG^37^ and not yet observed in CMG bound to DNA such as in the current structure, suggesting the “pumpjack” of N-tier/C-tier motion is less likely to be operational. Furthermore, a recent study of CMG–Pol ε bound to ATPγS at a DNA fork suggests that the C-tier AAA+ motor domains are planar rather than spiral, closing the gap at the motor domains of Mcm2 and 5^12,19^. The current CMG–forked DNA structure in the presence of ATP also shows a compact planar form at the C-tier (also observed in ref. ^19^, and thus we conclude that Pol ε is not required for the planar conformation observed in the presence of Pol ε^12,19^. Assuming that movement of the Mcm2 and 5 interface is incompatible with (or without) Pol ε binding, inch-worming translocation by movements between Mcm2 and Mcm5 may also be inoperable. Given the cell viability studies indicating all ATP sites play important roles in CMG function^35,36^, we do not exclude the staircasing model. Indeed, a recent report while this manuscript was under review analyzes the Drosophila CMG, showing several conformers during translocation, and proposes a model in which the DNA-binding loops of the C-domains function to pull the DNA through the central channel^38^.

In overview, the current report describes how DNA is unwound at the N-tier, by a dam-and-diversion process explaining how steric exclusion may function for CMG, while the DNA loops in the C-domain pull the leading ssDNA through the central channel.

## Methods

### DNAs

The DNAs used for these studies were the following oligonucleotides (Integrated DNA Technologies): a 45-mer lagging-strand oligo, (5′-GGCAGGCAGGCAGGCACACACTCTCCAATTA/iBiodT/CACTTCCTACTCTA-3′) and a 70-mer leading-strand oligo (5′-TAGAGTAGGAAGTGA/iBiodT/AATTGGAGAGTGTGTTTTTTTTTTTTTTTTTTTTTTTTTTTTTTTTTTT*T*T*T*T*T-3′). The asterisks indicate residues containing a non-bridging phosphothio linkage. The two oligos were annealed in equimolar amounts by heating to 90 °C followed by slow (1 h) cooling to room temperature. The hybrid was purified from a 8% native PAGE then sufficient streptavidin was added to saturate the DNA as predetermined in EMSA assays.

### Proteins

Mcm10 and CMG were purified^20^, with the following modifications. The Mcm10 contained a N-terminal hexahistidine tag and a C-terminal 3X FLAG tag. Briefly, 48 L *E. coli* cells carrying the Mcm10 T7-based *E. coli* expression vector were grown to OD 0.6 at 37 °C, then cooled to 15 °C and induced upon adding IPTG for an additional 8 h at 15 °C. The cells were harvested by slow speed centrifugation and lysed using a continuous flow high speed homogenizer. Cell debris was removed by centrifugation and applied to a 10 ml Chelating Sepharose Fast Flow column (GE Healthcare) charged with 50 mM NiSO_4_ in Buffer A (20 mM Tris–Cl pH 7.9, 5 mM imidazole, 500 mM NaCl, 0.01% NP-40). The column was washed with Buffer A, then eluted with 375 mM imidazole in Buffer A. The eluated material was applied to a 6 ml anti-FLAG M2 affinity gel (Sigma) equilibrated in Buffer B (20 mM Tris–Cl pH 7.5, 10% glycerol, 500 mM NaCl, 1 mM DTT, 1 mM MgCl_2_, 0.01% NP-40) and then washed with 20 column volumes of Buffer B + 1 M NaCl before eluting with Buffer B containing 0.2 mg/ml FLAG peptide (EZ Biolab, Carmel, IN, USA) using two 6 ml pulses of 20 min each and collecting 1.5 ml fractions. Eluted fractions were analyzed by SDS–PAGE, protein concentration was determined using Bradford Protein Stain (Sigma) wtih BSA as a standard. Proteins were then dialzed against 20 mM Tris–Cl pH 7.5, 10% glycerol, 50 mM NaCl, 1 mM DTT, 1 mM MgCl_2_) aliquoted, snap frozen in liquid nitrogen and stored at −80 °C.

The CMG–Mcm10 complex was reconstituted by mixing 765 pmol CMG with 3.1 nmol Mcm10 in 0.7 ml Buffer C (10 mM Tris–Cl pH 7.5, 200 mM KCl, 2 mM DTT, 2 mM MgCl_2_) for 30 min on ice. The mixture was then applied to a 0.1 ml MonoQ column, equilibrated in Buffer C. The column was washed using the same buffer and eluted with a 2.5 ml gradient of Buffer C from 0.2 M KCl to 0.6 M KCl. Fractions of 0.1 ml were collected, analyzed by Bradford and SDS–PAGE, pooled and dialyzed against 25 mM Tris–acetate pH 7.5, 50 mM K-glutamate, 2 mM Mg-OAc, and 1 mM DTT. Dialyzed material was analyzed again by Bradford stain for protein concentation, then aliquoted, snap frozen in liquid nitrogen and stored at −80 °C. An SDS–PAGE analysis of the CMG–Mcm10 complex used in this study is shown in Supplementary Fig. 8.

### Sample preparation for CryoEM

The replication fork bound to streptavidin (10 μM final) was added to a solution containing 1.2 mg/ml CMG–Mcm10 in 20 mM Tris–OAc pH 7.5, 40 mM K-glutamate, 40 mM KCl, 1 mM DTT, 2 mM Mg–OAc, along with 0.1 mM ATP. The sample was incubated for 5 min at room temperature, separated into 10 ml aliquots, snap frozen in liquid nitrogen, and stored at −80 °C. Samples were applied to cryo-EM grids immediately upon thawing.

### Cryo-EM

To prepare EM grids, 3 μl of CMG–Mcm10–bio-DNA-SA sample, at a final concentration of ~1.0 mg/ml, was applied to freshly glow-discharged holey carbon grids (C-flat 1.2/1/3). Grids were then incubated in 90% humidity for 10 s at 6 °C, blotted for 3 s and plunged into liquid ethane using a Thermo Fisher (TF) Vitrobot IV. Cryo-grids were loaded into a Titian Krios transmission electron microscope operated at 300 kV. Micrographs were collected automatically using low-dose mode at a nominal scope magnification of ×130,000 and a defocus range from 1.5 to 2.5 μm. A Gatan K2 summit direct electron detector was used for image recording under super-resolution mode, with an effective pixel size of 1.029 Å at the used magnification. The dose rate was 10 electrons per Å^2^ per second and the total exposure time was 8 s. The total dose was divided into a 40-frame movie, so individual frames were exposed for 0.2 s.

### Image processing and 3D reconstruction

Over 6000 raw movie micrographs were collected. Firstly, all the movie frames were aligned and superimposed by Motioncorr2^39^. Contrast transfer function parameters of each aligned micrograph were calculated with CTFFIND4^40^. We used RELION-2 for all the following image processing steps including particle autopicking, 2D classification, 3D classification, 3D refinement, postprocessing^41^. We manually picked about 5000 particles in different views to generate several 2D averages which were used as templates for automatic particle picking. Automatic particle picking was then performed for the whole data set. 718,903 particles were initially picked this way. These particles were then sorted according to the similarity to the 2D references; the bottom 10% of the particles that had very low *z*-scores were deleted from the particle pool. The 2D classification of all the remaining particles was performed and particles in “bad” classes (i.e. lack of defined CMG density features) were removed. 387,023 “good” particles with well-defined density features for the CMG structure were kept for the following 3D classification. We derived five 3D models from the dataset: two models had densities for the fork DNA and the particles associated with these maps were combined for further refinement; the other three models showed no DNA density or were distorted, and the particles associated with these three maps were discarded. A total of 162,550 particle images were used for further refinement, leading to the final 3D map with an estimated average resolution of 3.9 Å. The resolution estimations were based on gold-standard Fourier shell correlation calculations to avoid over-fitting and reported resolutions were based on the FSC = 0.143 criterion. The density map was corrected for the modulation transfer function of the detector and sharpened by applying a negative *B*-factor. Local resolution was estimated using ResMap^42^. These image processing and 3D reconstruction steps are illustrated in Supplementary Figs. 1–3.

### Atomic modeling, refinement, and validation

Models of all *S. cerevisiae* CMG subunits were extracted from the cryo-EM model of the yeast CMG–forked DNA (PDB ID 5U8S). DNA sequence was randomly assigned in the model. These models were rigid body fitted into the current 3D cryo-EM map with COOT^43^ and Chimera^44^. The individual chains of the CMG–DNA models were first refined as separate rigid bodies in the PHENIX program^45^, and subsequently rebuilt manually in COOT guided by residues with bulky side chains like Arg, Phe, Tyr, and Trp. The model was then refined in real space by phenix.real_space_refine and in reciprocal space by PHENIX with the application of secondary structure and stereochemical constraints. Because of the relatively weak density and limited resolution, the parent double-stranded DNA region was constrained to the ideal B form during refinement. The structure factors (including phases) were calculated by Fourier transform of the experimental density map with the program Phenix.map_to_structure_factors. The final models were validated using MolProbity^46^. Structural figures were prepared in Chimera and Pymol (https://www.pymol.org).

### Reporting summary

Further information on research design is available in the Nature Research Reporting Summary linked to this article.

## Supplementary information

Supplementary Information

Reporting Summary

Description of Additional Supplementary Files

Supplementary Movie 1

## Data Availability

The 3D cryo-EM map of CMG–forked DNA at 3.9 Å resolution has been deposited in the Electron Microscopy Data Bank under accession code EMD-20607. The corresponding atomic model has been deposited in the Protein Data Bank under accession code 6U0M. All relevant data are available from the authors.
