# Supplementary material for: DNA unwinding mechanism of a eukaryotic replicative CMG helicase
Source: Nat Commun. 2020 Feb 4;11:688. doi: 10.1038/s41467-020-14577-6 (PMC7000775; doi:10.1038/s41467-020-14577-6)
Supplement: Supplementary file 1 — Supplementary Information [file 41467_2020_14577_MOESM1_ESM.pdf]

**Supplementary Information for:**

# DNA unwinding mechanism of a eukaryotic replicative CMG helicase

**By Yuan et. al.**

# Supplementary Figures

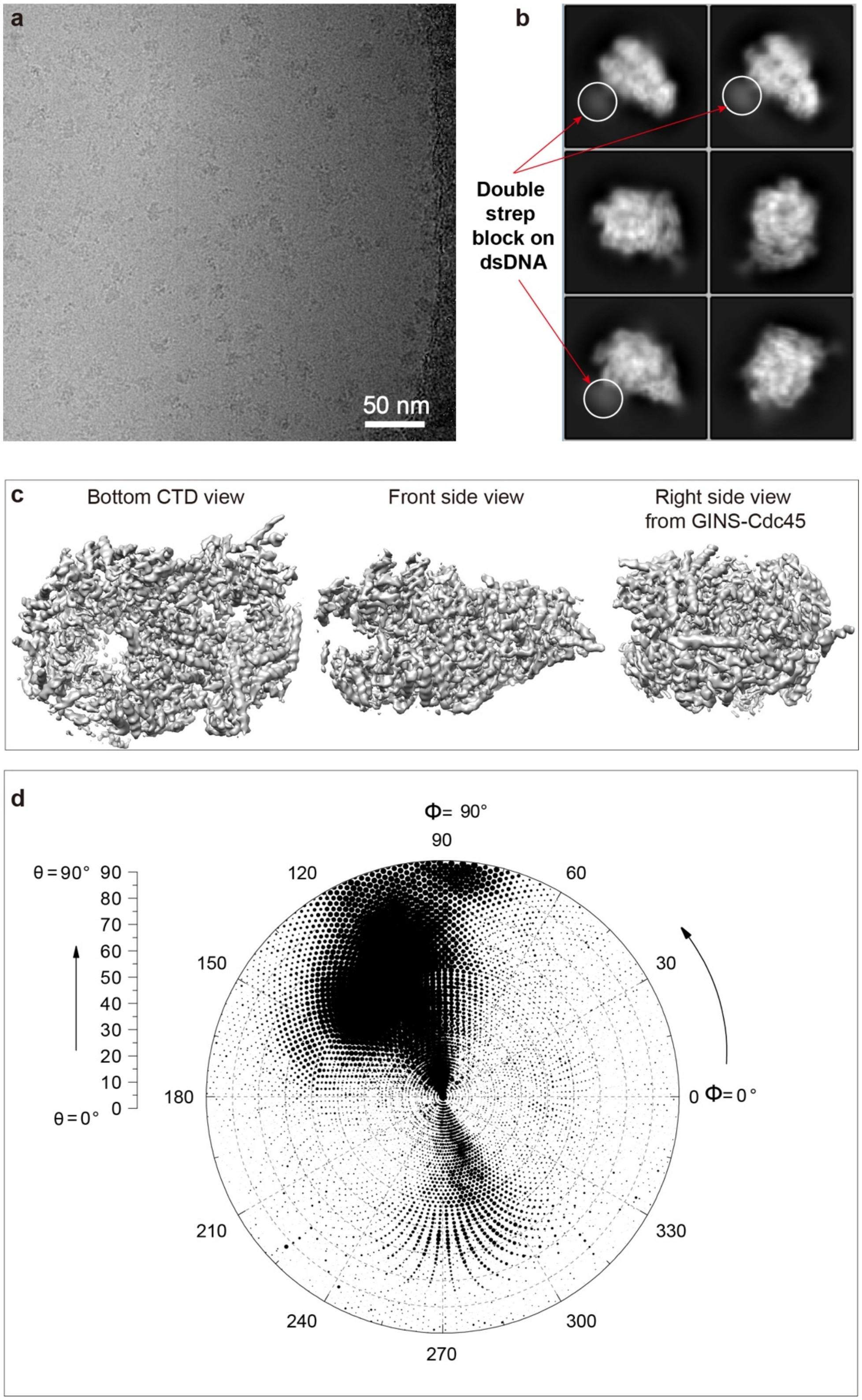


**Supplementary Figure 1. Cryo-EM of CMG bound to double-streptavidin blocked DNA fork in the presence of Mcm10 and ATP.** (**a**) A representative raw micrograph. (**b**) 2D class averages. (**c**) 3D map in different views. (**d**) Euler angle distribution of the particles in the final reconstruction.

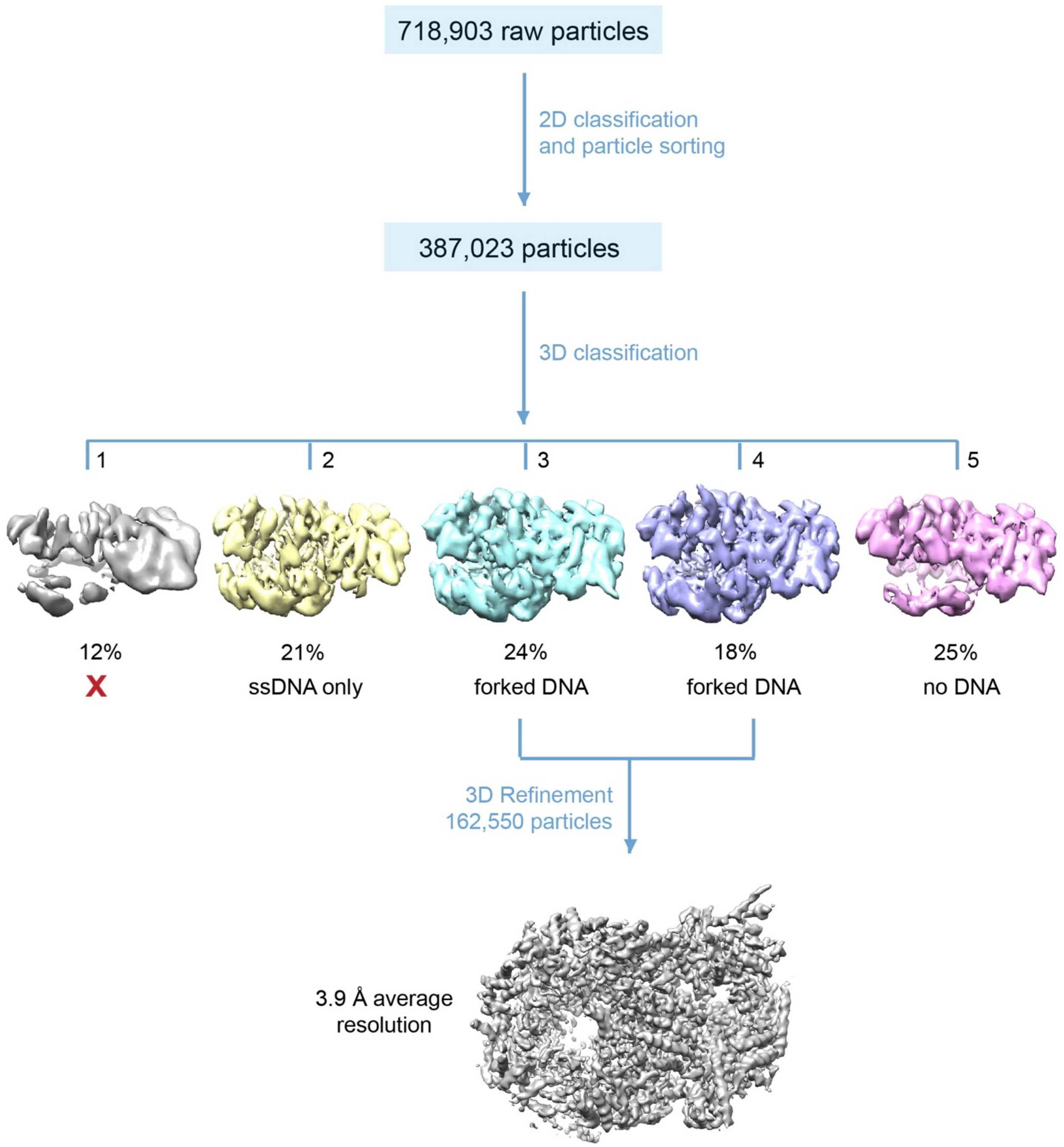


**Supplementary Figure 2. Image processing and 3D reconstruction procedure.** Raw particles from auto-picking were classified and those with clear class averages were selected for 3D classification. Among five 3D classes, only two contained forked DNA density. These two classes were selected for final refinement and 3D reconstruction, leading to the 3.9-Å resolution 3D map.

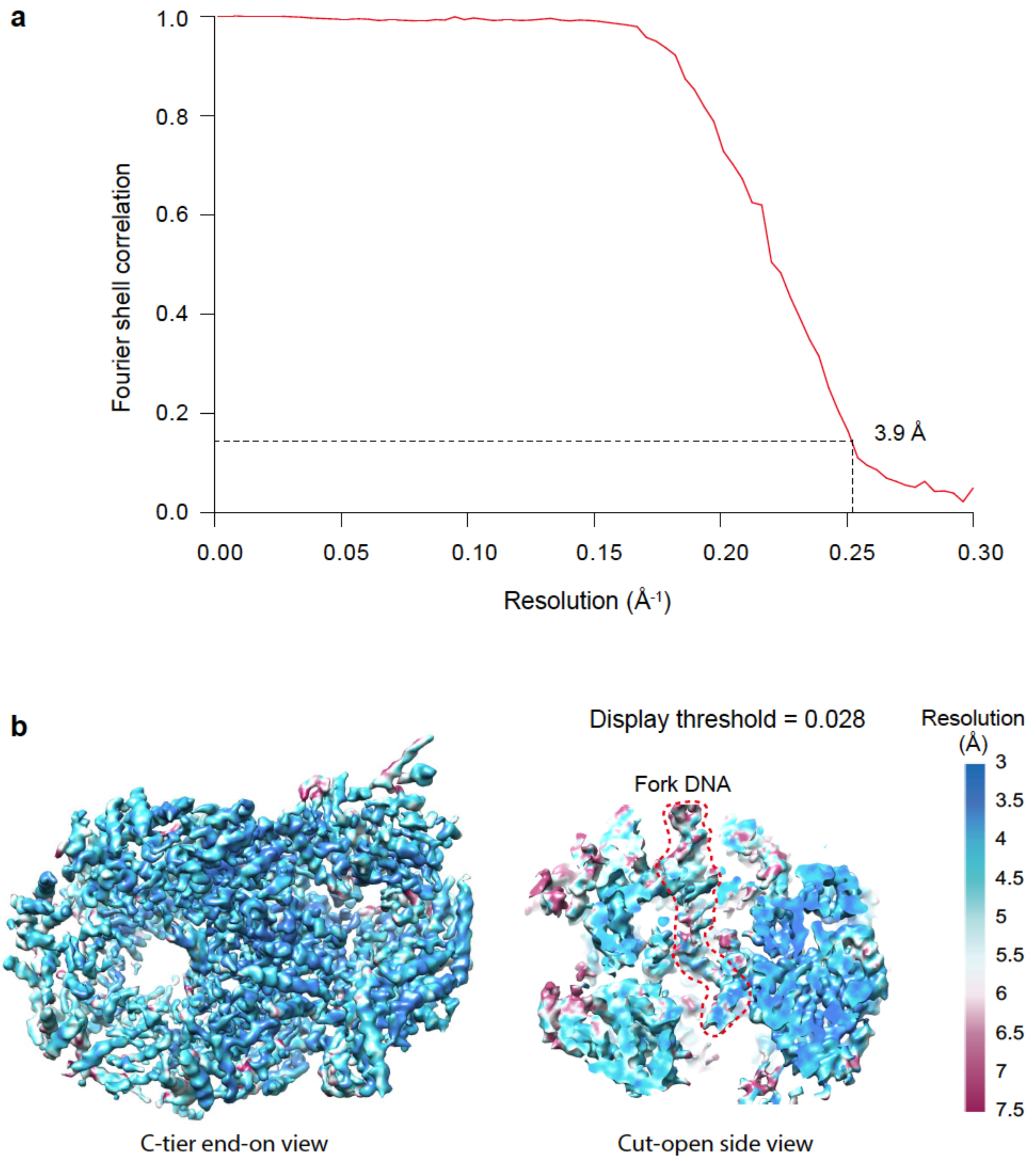


**Supplementary Figure 3. Resolution estimation of the final 3D map of the CMG–forked DNA.** (**a**) Gold standard Fourier shell correlation indicated an average resolution of 3.9 Å. (**b**) Local resolution maps. Left: Local resolution of the C-tier end-on view. Note that the parental dsDNA density is invisible at the raised display threshold. Right: A cut-open local resolution view of the DNA (dotted enclosure) and DNA binding regions inside the structure.

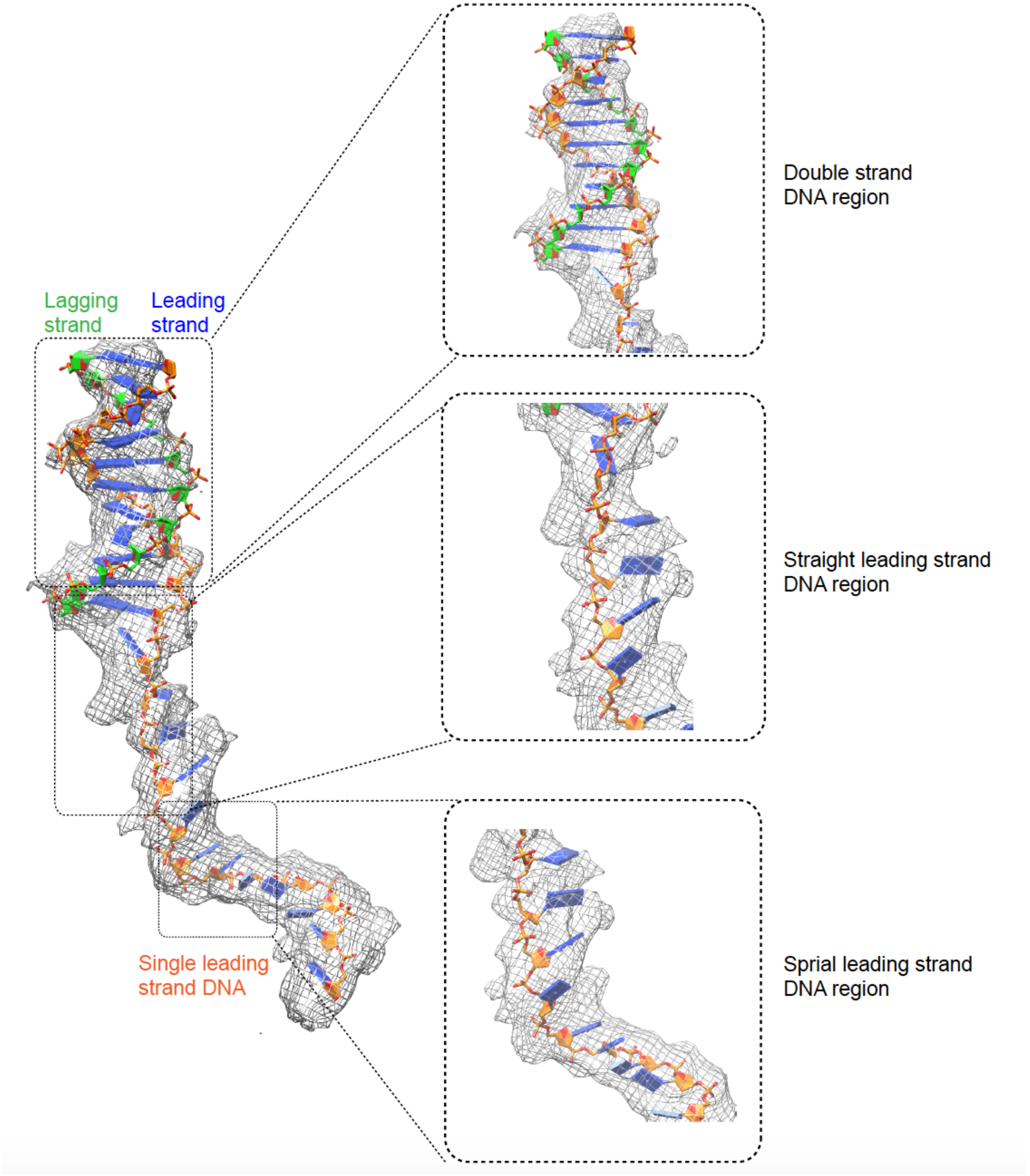


**Supplementary Figure 4. Surface-rendered cryo-EM 3D map of the forked DNA superimposed with the atomic model. Left:** Cryo-EM density of the forked DNA (mesh) and the atomic model. Insets are enlarged views of: top: the parental double-strand DNA region, middle: the straight linear leading ssDNA region, and bottom: the spiral leading ssDNA region. The display threshold for DNA density is 0.0275

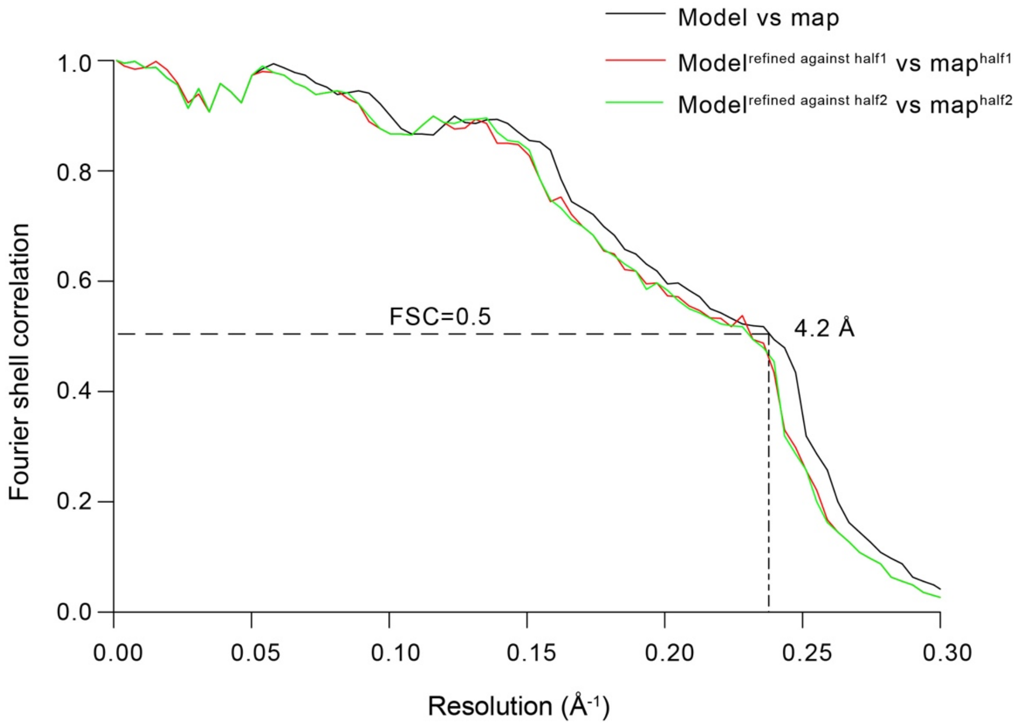


**Supplementary Figure 5. Map and atomic model correlation validation.** The atomic model is estimated to have an average resolution of 4.2 Å.

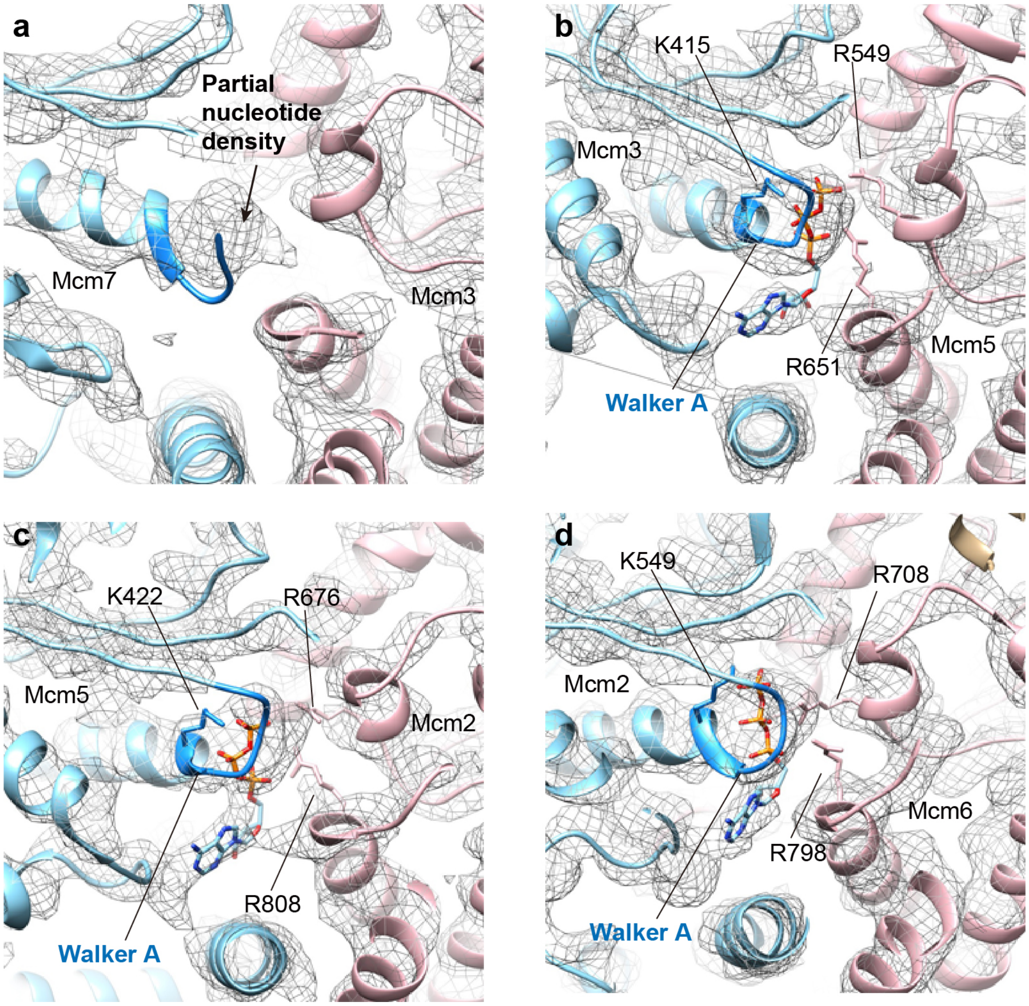


**Supplementary Figure 6. Nucleotide densities in the cryo-EM 3D map of CMG–forked DNA**. Only partial density is observed at Mcm7:Mcm3 interface (**a**). Nucleotide density with features most consistent with ATP is observed at the interface between Mcm3 and Mcm5 (**b**), between Mcm5 and Mcm2 (**c**), and between Mcm2 and Mcm6 (**d**). All densities are displayed at the same threshold of 3.5σ.

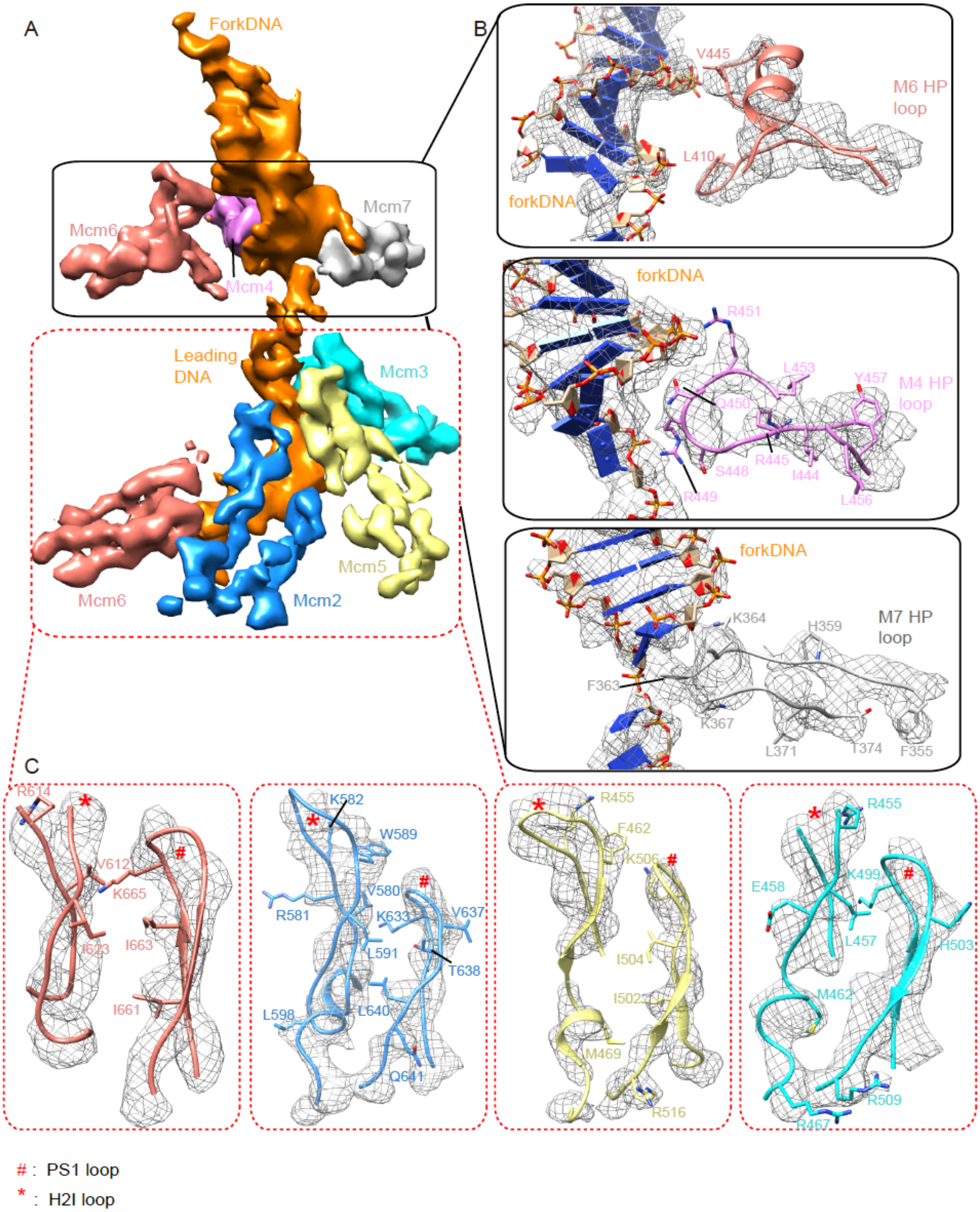


**Supplementary Figure 7. Modeling of CMG loops at the DNA fork junction and in the motor domains**. a) Density of OB hairpin (HP) loops in the N-tier of CMG subunits at the DNA forked junction. b) Modeling of OB hairpin (HP) loops at the forked junction in the subunits indicated. c) Modeling of the PS1 and H2i loops in the AAA+ C-tier subunits of CMG that interact with the single-strand DNA.

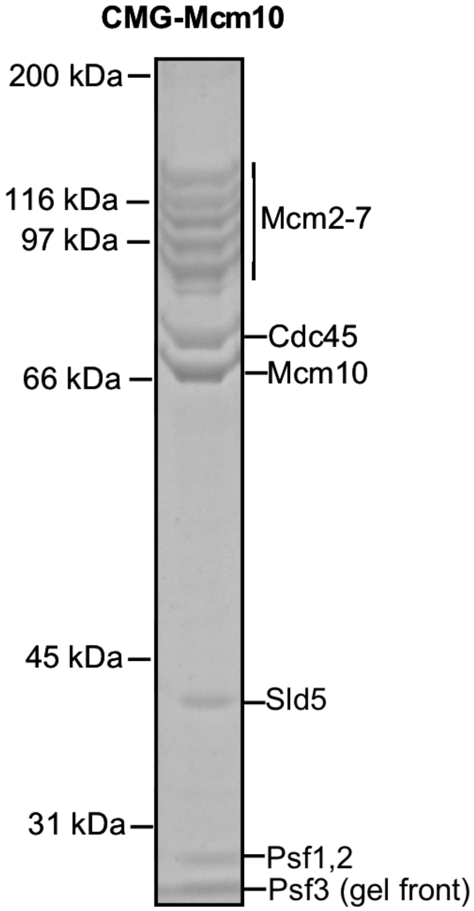


**Supplementary Figure 8. SDS PAGE gel of the CMG–Mcm10 sample used in this study**. The CMG–Mcm10 complex was reconstituted from CMG and Mcm10, then purified on a MonoQ column as described in Methods. The fraction used for this study, shown here, was analyzed in an 8% SDS PAGE. Size markers are shown to the left, and the identity of subunits is shown to the right.
