# Supplementary material for: DNA unwinding mechanism of a eukaryotic replicative CMG helicase
Source: Nat Commun. 2020 Feb 4;11:688. doi: 10.1038/s41467-020-14577-6 (PMC7000775; doi:10.1038/s41467-020-14577-6)
Supplement: Supplementary file 2 — Reporting Summary [file 41467_2020_14577_MOESM2_ESM.pdf]

nature research

Corresponding author(s): Michael O'Donnell

Last updated by author(s): Jan 8, 2020

Reporting Summary

Nature Research wishes to improve the reproducibility of the work that we publish. This form provides structure for consistency and transparency in reporting. For further information on Nature Research policies, see Authors & Referees and the Editorial Policy Checklist .

Statistics

For all statistical analyses, confirm that the following items are present in the figure legend, table legend, main text, or Methods section.

n/a	Confirmed	
☐	☒	The exact sample size (n) for each experimental group/condition, given as a discrete number and unit of measurement
☐	☒	A statement on whether measurements were taken from distinct samples or whether the same sample was measured repeatedly
☒	☐	The statistical test(s) used AND whether they are one- or two-sided<br>*Only common tests should be described solely by name; describe more complex techniques in the Methods section.*
☒	☐	A description of all covariates tested
☒	☐	A description of any assumptions or corrections, such as tests of normality and adjustment for multiple comparisons
☒	☐	A full description of the statistical parameters including central tendency (e.g. means) or other basic estimates (e.g. regression coefficient) AND variation (e.g. standard deviation) or associated estimates of uncertainty (e.g. confidence intervals)
☒	☐	For null hypothesis testing, the test statistic (e.g. F, t, r) with confidence intervals, effect sizes, degrees of freedom and P value noted<br>*Give P values as exact values whenever suitable.*
☒	☐	For Bayesian analysis, information on the choice of priors and Markov chain Monte Carlo settings
☒	☐	For hierarchical and complex designs, identification of the appropriate level for tests and full reporting of outcomes
☒	☐	Estimates of effect sizes (e.g. Cohen's d, Pearson's r), indicating how they were calculated

Our web collection on statistics for biologists contains articles on many of the points above.

Software and code

Policy information about availability of computer code

Data collection	SerialEM in Titan Krios and EPU as implemented in Arctica by the manufacturer Thermo-Fisher Scientific.
Data analysis	RELIONS 2.0, MotionCorr, CTFFIND4, Chimera, Pymol, Coot, Phenix, and MolProbity.

For manuscripts utilizing custom algorithms or software that are central to the research but not yet described in published literature, software must be made available to editors/reviewers. We strongly encourage code deposition in a community repository (e.g. GitHub). See the Nature Research guidelines for submitting code & software for further information.

Data

Policy information about availability of data

All manuscripts must include a data availability statement. This statement should provide the following information, where applicable:

- Accession codes, unique identifiers, or web links for publicly available datasets
- A list of figures that have associated raw data
- A description of any restrictions on data availability

The 3D cryo-EM maps of CMG-forked DNA at 3.9 Å resolution has been deposited in the Electron Microscopy Data Bank under accession code EMD-20607. The corresponding atomic model has been deposited in the Protein Data Bank under accession code PDB-6U0M.

Field-specific reporting

Please select the one below that is the best fit for your research. If you are not sure, read the appropriate sections before making your selection.

Life sciences study design

All studies must disclose on these points even when the disclosure is negative.

Sample size	We collected over 6,000 raw movie micrographs, from which 718,903 raw particles were initially picked. After data exclusion as described below based on 2D and 3D classification analyses, a final dataset of 162,550 particles were retained for final 3D reconstruction and refinement, leading to the reported 3D density map at an average resolution of 3.9 Å resolution.
Data exclusions	"Bad" raw particles that did not produce good 2D class averages with structural details were excluded in the first round of particle selection. In the second round, particles that did not belong to 3D class maps with defined features were excluded. These are per standard image processing practice. In the final round of selection, particles that belonged to 3D classes that did not contain forked DNA density were also excluded, because our goal was to determine structure of CMG helicase bound to the forked DNA.
Replication	Reproducibility resides in the large number of particles used to derive at the final 3D maps or 2D averages. The reliability and the resolution is measured by gold-standard Fourier shell correlation.
Randomization	The raw particles were randomly selected by computer program (RELIONS 2.0).
Blinding	The investigators were blinded to the specific data points during data collection and analysis.

Reporting for specific materials, systems and methods

We require information from authors about some types of materials, experimental systems and methods used in many studies. Here, indicate whether each material, system or method listed is relevant to your study. If you are not sure if a list item applies to your research, read the appropriate section before selecting a response.

Materials & experimental systems

n/a	Involved in the study	
☒	☐	Antibodies
☒	☐	Eukaryotic cell lines
☒	☐	Palaeontology
☒	☐	Animals and other organisms
☒	☐	Human research participants
☒	☐	Clinical data

Methods

n/a	Involved in the study	
☒	☐	ChIP-seq
☒	☐	Flow cytometry
☒	☐	MRI-based neuroimaging
