# Supplementary material for: DNA unwinding mechanism of a eukaryotic replicative CMG helicase
Source: Nat Commun. 2020 Feb 4;11:688. doi: 10.1038/s41467-020-14577-6 (PMC7000775; doi:10.1038/s41467-020-14577-6)
Supplement: Supplementary file 3 — Description of Additional Supplementary Files [file 41467_2020_14577_MOESM3_ESM.pdf]

Description of Additional Supplementary Files

File Name: Supplementary Movie 1
Description: CMG-forked DNA and OB fold loops. The video shows rotation of the CMG-forked DNA complex structure rendered in cartoon and colored according to individual chains as in the main text figure. The OB fold hairpin loops that interact with the forked junction are noted by arrows.
